# The reduction in anemia through normative innovations (RANI) project: study protocol for a cluster randomized controlled trial in Odisha, India

**DOI:** 10.1186/s12889-020-8271-2

**Published:** 2020-02-07

**Authors:** Hagere Yilma, Erica Sedlander, Rajiv N. Rimal, Ichhya Pant, Ashita Munjral, Satyanarayan Mohanty

**Affiliations:** 1 Department of Prevention and Community Health, George Washington University Milken Institute School of Public Health, Washington, District of Columbia USA; 2 Department of Health, Behavior and Society, Johns Hopkins University Bloomberg School of Public Health, Baltimore, MD USA; 3 Department of Social and Economic Empowerment, IPE Global Limited, New Delhi, Delhi India; 4 DCOR Consulting, Bhubaneswar, India

**Keywords:** Social norms, Iron deficiency anemia, Cluster randomization, Controlled trial

## Abstract

**Background:**

More than half of women in India are anemic. Anemia can result in fatigue, poor work productivity, higher risk of pre-term delivery, and maternal mortality. The Indian government has promoted the use of iron-folic acid supplements (IFA) for the prevention and treatment of anemia for the past five decades, but uptake remains low and anemia prevalence high. Current programs target individual-level barriers among pregnant women and adolescents, but a more comprehensive approach that targets multiple levels among all women of reproductive age is needed to increase uptake of IFA and iron-rich foods.

**Methods:**

The Reduction in Anemia through Normative Innovations (RANI) project is a norms-based intervention to reduce anemia among women of reproductive age. We will evaluate the intervention through a clustered randomized controlled trial in Odisha, India. We will collect data at three time points (baseline, midline, and end line). For the study, we selected 89 clusters of villages, which we randomized into treatment and control on a 1:1 basis. The treatment arm will receive the RANI project components while the control arm will receive usual care. Fifteen clusters (40–41 villages) were selected and 4000 women (2000 in each arm) living in the selected clusters will be randomly selected to take part in data collection. Women in both study arms will have their hemoglobin concentrations measured. They will also complete in-person surveys about their knowledge, attitudes, perceptions of iron folic acid supplements, and nutritional intake. We will also select a smaller cohort of 300 non-pregnant women (150 in each arm) from this cohort for additional physical activity and cognitive testing. We will conduct both within- and between-group comparisons (treatment and control) at baseline, midline and end line using t-tests. We will also conduct structural equation modeling to examine how much each factor accounts for IFA use and hemoglobin levels.

**Discussion:**

This RCT will enable us to examine whether a social norms-based intervention can increase uptake of iron folic acid supplements and iron rich foods to reduce anemia.

**Trial registration:**

This trial was registered with Clinical Trial Registry- India (CTRI) (CTRI/2018/10/016186) on 29 October 2018.

## Background

Anemia is a serious health concern in India, where more than half of women of reproductive age (WRA) are anemic [1]. It is mostly associated with fatigue and thus poor work productivity [2], but if left untreated, anemia can lead to poor birth outcomes, including higher risk for preterm delivery and maternal mortality [3]. Anemia during pregnancy can also inhibit physical and cognitive development in children [4–6].

In Odisha, India (the site of this study) the majority of anemia cases are a result of iron-deficiency, due to poor dietary iron intake, low iron absorption, and iron-loss during intestinal worm infection, pregnancy and menstruation. As one of six Global Nutrition Targets for 2025, the World Health Organization (WHO) has set forth a series of recommendations to prevent and reduce anemia [7]. Among these recommendations is regular iron-folic acid (IFA) supplementation for all women of reproductive age between 15 and 40 years old (including pregnant and non-pregnant women) in regions where more than 40% of women are anemic [7].

India has implemented several national-level programs to increase IFA supply over the last 50 years. However, anemia levels remain high, partly because of the scarcity of interventions to improve the demand for and uptake of IFA and iron-rich foods. Of late, efforts to promote IFA consumption in India have adopted a life cycle approach by including women of reproductive age (non-pregnant and non-lactating) for IFA supplementation rather than exclusively targeting pregnant and lactating women, adolescents and/or children [8, 9]. Unlike pregnant women, non-pregnant and out-of-school women are poorly served as the government is currently in the process of rolling out its IFA supplementation strategy to these important sub-populations. Not surprisingly, adherence rates in this group is also poorly understood. For example, the Indian National Family Health Survey (NFHS) collects data on IFA adherence only for pregnant women [1]. Nevertheless, available data indicate that adherence is poor as only 30.3% of mothers in India reported consuming IFA for 100 days or more when they were pregnant, although 91% percent received IFA [1]. To effectively reduce anemia in India, both pregnant and non-pregnant WRA should not only receive IFA, but they should also take it regularly.

Innovative approaches that increase IFA demand can propel changes at multiple levels (individual, interpersonal, community and policy). Shet et al. [10] demonstrated that educational counseling delivered to mothers and caregivers can improve IFA consumption and reduce anemia in children. Behavior change interventions that target the individual directly are also effective in improving IFA consumption. Adolescent girls in Delhi showed improvement in their IFA consumption, along with their knowledge and attitudes around IFA and anemia, after receiving an educational intervention delivered in schools [11]. Many IFA-focused interventions in India that target adolescent school girls have also been successful in reducing anemia prevalence through supplementing IFA provision with educational information [12]. While programs of this sort for adult women are limited, a similar communication intervention delivered through women’s Self Help Groups to pregnant women in rural India was effective in improving IFA consumption among other pregnancy-related behaviors [13]. While the vast majority of behavior change interventions that promote IFA consumption target in-school girls or pregnant women, they should be extended and adapted for all WRA, regardless of pregnancy or school status.

The Reduction in Anemia through Normative Innovations (RANI) Project aims to reduce the burden of anemia among all WRA in India through a social norms-based approach. Social norms are based on the idea that people conform to the behaviors they perceive others around them are engaging in. Thus, the extent to which WRA believe others are taking IFA can influence their own IFA consumption. The theoretical underpinnings of the intervention are described in greater detail in later sections. In this paper, we describe the randomized control trial protocol, which we are using to test the efficacy of the RANI Project in increasing IFA and iron rich food consumption to reduce anemia among WRA.

### Objectives

The objective of this study is to investigate the ability of a norms-based behavior change intervention to reduce anemia among women of reproductive age in Odisha, India. We will test the following hypotheses:

H1. Changes in women from baseline to end line in the intervention arm will be significantly greater than corresponding changes in the control arm in the following outcomes: (a) anemia status, (b) IFA use, (c) mental health/depression, (d) physical activity (e) work capacity, (f) consumption of iron rich foods and (g) cognitive functioning;

H2. Social norms serve as a mediator in the relationship between intervention exposure and study outcomes; and.

H3. Changes in women baseline to end line in the intervention arm will be significantly greater than corresponding changes in the control arm in knowledge, attitudes, perceptions, consumption of iron-rich foods, and use of IFA.

## Methods

### Study setting

We will conduct the study in Odisha, which is on the eastern coast of India, where 83% of residents live in rural areas. Across Odisha, 94% of households are Hindu and 23% belong to a tribal culture. Around three fourths (73%) of the total population and nearly two-thirds (64%) of women in Odisha are literate [14]. Additionally, the total fertility rate (TFR) is approximately 2.1 children per woman in Odisha. Around half of WRA in Odisha are anemic (51.0%). The prevalence of anemia among women is high across subgroups: those who are breastfeeding (54.8%), pregnant (47.6%), and WRA who are neither breastfeeding nor pregnant (50.3%). Women with less education and who belong to Scheduled Tribes are more likely to be anemic [1].

Within Odisha, we chose Angul district for our study site because its anemia prevalence is similar to that of the state and the rest of India [1]. Anemia rates and IFA adherence in Angul also follow a similar pattern to Odisha State: 44.0% of the Angul population is anemic and only 38% of women consume IFA when they were pregnant. We selected two blocks within the Angul district, Athmallik and Kishorenagar, as study sites (a block is the administrative unit larger than a village but smaller than a district and each block encompasses several villages). The two blocks were not randomly selected, rather they were selected because they are adjacent to each other, spread over an area of 1278 sq. kilometers (499 sq. miles) and are representative of the district. According to the 2011 census, the two blocks have a total of 588 villages, accounting for a total of 218,373 people in 50,463 households [14]. In the two blocks, nearly one fourth of people are tribal, a third are literate, and about half of women work outside of the home.

### Design

We will use a cluster randomized controlled trial (RCT) design. In this design, villages will be randomized on a 1:1 ratio to receive the treatment or continue with usual care (defined as the currently existing and ongoing efforts to reduce anemia in Odisha). Treatment is defined as exposure to one or more components of the RANI project. As this is a community-level intervention, we used a cluster design to prevent contamination across communities.

We grouped together villages into clusters of 1–4 villages, resulting in eighty-nine total clusters. A geographical buffer of at least one village or natural structure (e.g., a mountain) was maintained between clusters to limit contamination. We first used a random number generator to randomly assign clusters into treatment (*k* = 50 clusters) and control arms (*k* = 38 clusters). Clusters were randomly given a value of ‘1’ or ‘2’; those that were given a ‘1’ were assigned to the treatment and those that were given a ‘2’ were assigned to the control. Thus, the number of clusters in each arm are not exactly equal.

**Fig. 1:**
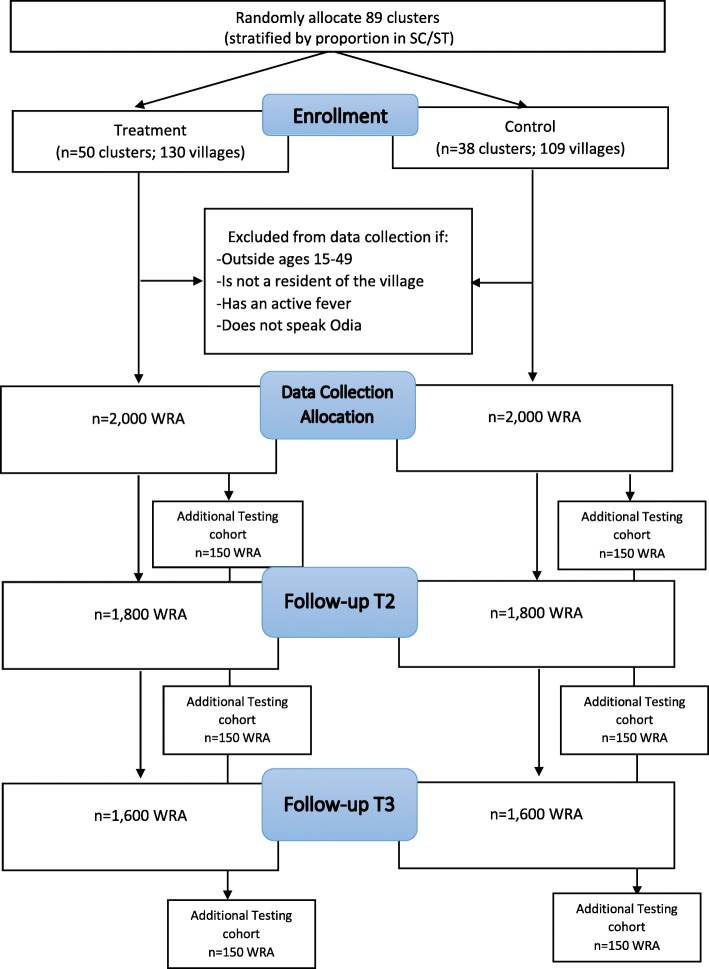
Custer Randomized Control Trial Schema

We then segmented clusters by proportion of minority populations (in India, they are called scheduled castes and scheduled tribes) and then selected three clusters from each stratum for data collection so that 15 clusters (which comprised 41 villages) from the treatment arm and 15 (comprising 40 villages) from the control arm were selected for data collection. The decision to select a smaller subset of 15 clusters from each arm for data collection was made in order to maximize the sample size per cluster. Data collectors and program implementers will be blinded with regard to treatment and control status of villages. Data collection will occur at three time points: baseline, midline, and end line. The overall schema of the study design is depicted in Fig. 1.

### Participants

All members of the treatment clusters meeting the inclusion criteria will be eligible to participate in the trial.

#### Inclusion criteria

All women selected for data collection must be between 15 and 49 years old, a resident of the village, and speak Odiya. Additionally, as this is a longitudinal study, participants must indicate that they are not planning to move out of the village for the next two years.

#### Exclusion criteria

We will exclude women with an active fever at data collection and refer them to the closest health center, as the interview may take up to an hour or longer and may exacerbate any illness they may already have. Once excluded from baseline, the woman will no longer be eligible for data collection and midline and endline. However, women who are excluded from data collection may still be reached in the intervention if they live in a treatment village. We will also refer those with severe anemia to the local health center, but they will *not* be excluded from data collection. Though pregnancy status is not an inclusion or exclusion criterion, we will exclude currently pregnant women from certain components of data collection that could put them at risk —they will only take part in the survey and provide hemoglobin measurements.

### Rational and overview of the intervention

We developed the intervention based on the literature as well as findings from our formative research to understand barriers to and facilitators of IFA use. The formative research [15] comprised the following components:

- 16 focus group discussions with women of reproductive age, their husbands, and mothers-in-law
- 25 key informant interviews with self-help group leaders, medical doctors, teachers, natural healers, and frontline health workers
- A perceptual mapping exercise to understand how women of reproductive age, their mothers-in-law, and their husbands conceptualize IFA and other anemia-related items (e.g., fatigue, fruits and vegetables, medical care, etc.)

#### Formative research results

The formative research provided insights at multiple levels. At the *individual level*, we learned that the majority of people had basic knowledge about anemia and knew that IFA can prevent and treat anemia. However, women did not have a clear understanding about their own anemia risk; rather, they had normalized the existence of milder forms of anemia. We also identified both real and perceived side effects of IFA use, including some misperceptions.

At *the interpersonal level*, we found that perceptions of approval from referent groups (i.e., husbands and mothers-in-law) played a major role in women’s decisions to take IFA. These referent groups, largely mothers-in-law, were also found to perpetuate misconceptions around IFA use, including the belief that taking IFA during pregnancy would result in an abnormally large baby during and thus complicating delivery.

At the *community level*, we found that women’s health was not a priority and women were expected to take care of their families before thinking about their own well-being. They were also expected to work for the household all day, leaving little time for themselves, thus reducing their ability to visit a health center to get tested for anemia or to obtain IFA.

At the *policy level*, we found that out-of-school non-pregnant women were not directly served by existing government practices of delivering IFA. Health workers distribute IFA to pregnant women in their homes and in village health and nutrition days, and adolescents obtain them in schools; non-pregnant women do not know that they should be taking IFA weekly (per Indian government guidelines) and therefore do not seek it out [8, 9, 16].

#### The role of social norms

The proposed intervention will use a social norms approach to incorporate factors at play at multiple levels of the socio-ecological model. Social norms are based on the idea that people change behaviors because they perceive that others around them are changing and they do not want to be left behind. *Descriptive norms* refer to people’s perceptions about the prevalence of a behavior – what they believe others are doing – and *injunctive norms* refer to pressures people feel to conform [17]. Additionally, *collective norms* refers to the actual prevalence of behavior among one’s peers (e.g., the actual number of women taking IFA in a woman’s village) [18].

#### Theoretical underpinning

The RANI project intervention is based on the theory of normative social behavior (TNSB), which posits that social norms drive behavior and that this influence is further heightened when moderators are in favor of the behavior [19]. According to the TNSB, the relationship between social norms and behavior is moderated by a number of factors, including behavioral (e.g., access and outcome expectations), individual-level (e.g., self-efficacy, knowledge, and risk perception), and contextual-level (e.g., interpersonal discussion, gender norms, and nutrition) factors. Following the theoretical guidelines, this project will focus on descriptive norms (perceived prevalence), injunctive norms (pressures people feel to conform), and collective norms (actual prevalence) surrounding IFA consumption.

**Fig. 2:**
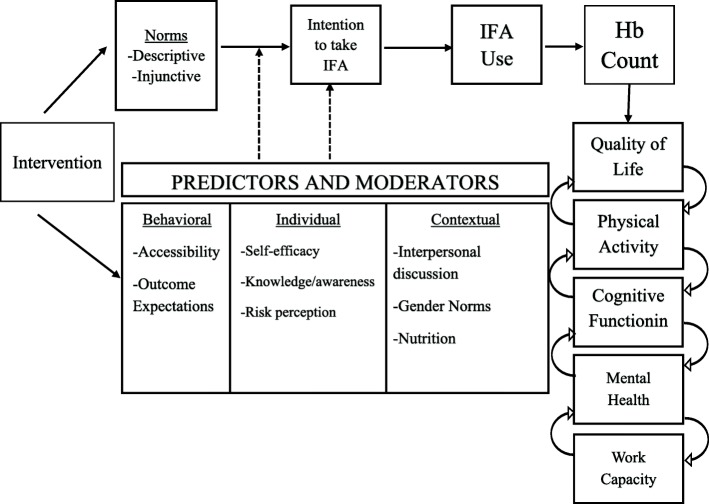
Theory of Change

The TNSB also posits that norms, by themselves, may not be enough to propel change [20]; normative information often must be coupled with information about benefits of performing the behavior [21], the behavior itself must be easy to enact [22], and people must be convinced that others in their social network are also engaging in the behavior [23]. Thus, if people learn that others in their social network are taking IFA, that they themselves can also take them, and that these supplements have benefits (e.g., improving their health or providing them with more energy), they may be persuaded to do the same. The overall theory of change for the intervention can be found in Fig. 2**.** The consideration of the potential moderators that can propel norms into action can help combat attributable barriers of IFA consumption, such as unpleasant side effects. For example, we know from our formative research that women prioritize their ability to help their family. Guided by TNSB, we suspect that when positive descriptive norms around IFA (i.e., the belief that other WRA are taking IFA) are coupled with positive injunctive norms (i.e., perceptions of support from their mother-in-laws and husbands) and strong risk perceptions and other psychosocial factors related to anemia and IFA, norms may translate into IFA consumption despite the barriers related to side effects.

Our approach will focus on generating demand at multiple levels. At the individual level, we will raise awareness and knowledge around anemia, correct misperceptions about the role of iron (in making deliveries more difficult), increase risk perception (susceptibility and severity), and enhance self-efficacy. At the interpersonal level, we will promote positive social norms around taking IFA and eating iron rich foods, along with other foods that promote iron absorption. We will focus on improving descriptive norms through demonstration events in communities in which women take IFA in a public setting and where community-level hemoglobin counts are graphically displayed. To improve injunctive norms, we will focus on persuading women’s husbands and their mothers-in-law to support them taking IFA. At the policy level, we will engage with health officials at multiple levels and policymakers at the state level to ensure that they are promoting IFA guidelines, that there is a continuous supply of IFA, and that they are promoting demand-generation efforts. A description of all RANI activites can be found in Table 1, along with the timeline for the intervention (Table 2).

**Table 1:** RANI Intervention Activities

Intervention Component	Description	Objectives	Dose	Delivered by
T4 Sessions and Community Engagement Meetings	10 Participatory Learning and Action (PLA) sessions and community engagement meetings developed in collaboration with our implementation partner and another local partner specializing in the methodology based in Delhi. Sessions and community engagement meetings include a mix of didactic learning and games focused on specific topics related to anemia prevention and theoretical constructs. Target population: women of reproductive age (WRAs), mothers-in-law, husbands, frontline workers, and government officials/policy makers	The sessions will cover information related to anemia, knowledge and awareness about iron folic acid (IFA) supplements, dietary diversity, social norms, malaria, water and sanitary hygiene (WASH), and deworming.	Monthly	RANI Community Facilitators
Rani Comm	6 media products – 3-4 min locally shot videos complimenting the concept developed in collaboration with our implementation partners and a local media production house based in Delhi. We show videos to small groups on smart phones. In year three, we will have evening viewings on large projectors in each village. Target population: women of reproductive age (WRAs), mothers-in-law, and husbands	Each video highlights the key messages of the program (including modeling positive social norms around IFA) and addresses the myths and barriers that we identified during the formative research around anemia/IFA consumption.	Ongoing basis	RANI Community Facilitators
Hemoglobin Testing and Demonstration	Fifteen women from each of the 130 intervention villages will undergo monthly hemoglobin testing in their village (*n* = 1950 women per month). The testing is user-friendly with instant digital results. Their Hemoglobin levels along with their IFA consumption status will also be tracked on a monthly basis. We designed cards with different colors indicating anemia severity, (green, yellow, orange, red), along with relevant behavioral nudges to share the Hemocue results. We use individual tracking cards to monitor hemoglobin progress. We share results at the individual, group, and inter-village level to trigger demand for IFA uptake and consumption of iron rich foods. Target population: women of reproductive age (WRAs) for testing and their families/villages for demonstrations	The goal of this activity is to promote three types of feedback – ipsative (comparisons between community hemoglobin levels in the past and the present), social (how two neighboring communities are faring, compared to participants’ results at the individual, group, and inter-village level to trigger demand for IFA uptake and consumption of iron rich foods.	Monthly	RANI Community Facilitators
mRANI	mRANI or mobile-RANI is a smaller intervention built into the larger RANI trial to increase demand and adherence to IFA supplements using interactive norms-based audio messages. After midline data collection (Spring 2020), we will begin the 12-week intervention. We will send audio messages via automated phone calls. As this is an interactive dialogue, we will encourage participants to ask questions, seek additional information, share their experience, or provide feedback via text or phone call. We will use an open-source two-way Interactive Voice Response (IVR) system. We chose audio recordings to be able to reach women with low literacy in an approachable, efficient, and cost-effective way. Target population: women of reproductive age (WRAs)	The primary objective of mRANI is to examine the effectiveness of automated voice call messages with a social-norms framing to increase IFA demand among women living in low-resource settings. Each call will be 30 s long and enrolled women will receive two calls per week.	12 weeks between midline and endline assessments	Open-source Interactive Voice Response Software (IVRS)

**Table 2:**
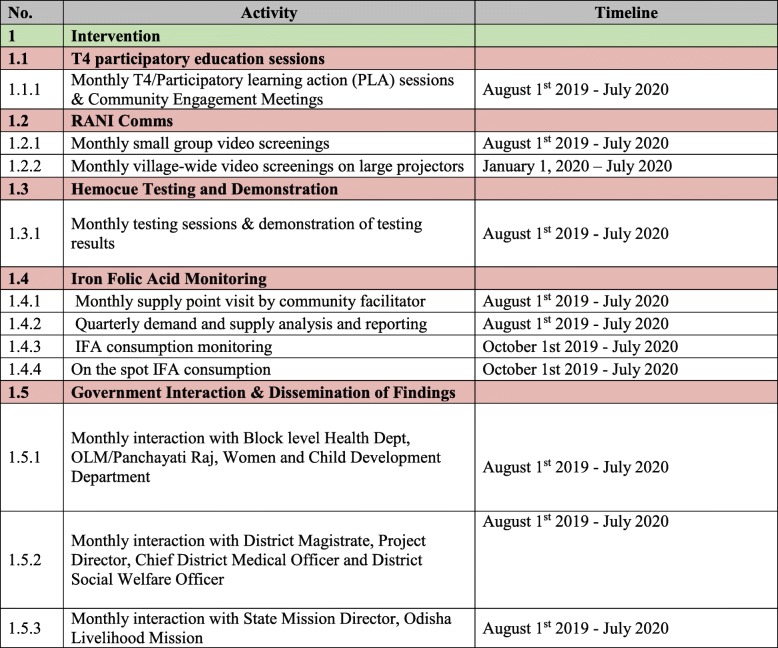
RANI Intervention Timeline.

To catalyze individual-level change, the intervention will use a T4 approach: *Train, Tell, Test, and Tweak*. We will *train* WRA and other community members through self-help group (SHG) meetings about anemia, IFA, and iron-rich foods so they can bring this knowledge to their community. SHGs are the primary platform of women’s empowerment across India. Within each village, several SHGs convene regularly to empower women with financial literacy and other forms of support. The involvement of SHGs in the intervention comes through our partnership with the Odisha Livelihood Mission (OLM), the government organization responsible for the formation and management of women SHGs in the state. We will develop ten modules that will include a mix of didactic learning and games focused on specific behavior changes and then use the SHG platform to conduct follow-up sessions in small groups.

The intervention will also *tell* the stories of overcoming barriers to IFA use through six short videos that feature members of the target audiences (WRA, husbands, mothers-in-law, and frontline workers) overcoming the barriers that we identified in the formative research. We will show the videos during SHG meetings, village health and nutrition days, and community festivals. The goal of the videos is to promote collective interest around anemia prevention by increasing knowledge, improving risk perception, enhancing perceptions of efficacy, and promoting positive social norms. We will also send regular voice-based messages to mobile phones to remind women to take the IFA, and we will also reinforce social norms around taking them.

We plan to *test* WRA both in the SHGs and throughout the community for anemia via a point-of-care hemoglobin test. We will then display the individual- and SHG- level results in the community, using graphic methods appropriate for low-literacy audiences.. The goal of this activity is to promote three types of feedback – ipsative (comparisons between community hemoglobin levels in the past and the present), social (how two neighboring communities are faring, compared to participants’ own community), and aspirational (how the community is faring, compared to goals set by the community early on).

Based on continuous monitoring and evaluation, we will *tweak* the curriculum, messages, and/or overall approach. We will gather real-time quantitative and periodic qualitative data about each intervention component to ensure fidelity and to gather feedback about which areas are working and which areas need improvement. The qualitative data will also capture unintended consequences (both positive and negative) as a result of the intervention.

### Outcomes

The primary evaluation outcome is anemia among women of reproductive age, defined as having hemoglobin count less than 12 g/dcl among non-pregnant women and less than 11 g/dcl among pregnant women. We will measure this via a HemoCue point-of-care blood prick. We will also measure self-reported IFA use via a tablet-based survey.

Several secondary outcomes will also be measured in all participants to understand the mechanism of change, including: (a) knowledge and perceptions about anemia and IFA, (b) social norms, (c) diet, (d) mental health, and (e) quality of life. In a smaller sample of non-pregnant women, we will assess other secondary outcomes, including (a) physical activity (through ActivPal readings), (b) work capacity (through the modified Queens College step test), and (c) socio-cognitive functioning (through paper and computer-based response time tasks).

### Recruitment

Within the selected clusters for data collection (described above), women between the ages of 15 and 49 residing in treatment (*n* = 2000) and control (*n* = 2000) clusters will be randomly selected and recruited to participate in the impact evaluation. Sampling will be stratified by treatment/control, village size and household.

To do so, we will create a household listing of eligible women within the selected clusters. The sampling size from each cluster will be proportional to population so that 60% of women in each arm come from high-population areas, 30% come from medium population areas, and 10% come from low population areas. Once we determine the number of eligible women, we will sample every n^th^ household to get our total sample.

As mentioned and shown in Fig. 2, the sampling design also includes a greater-intensity subset of participants from which certain secondary outcomes will be measured (i.e., physical activity, work capacity, and socio-cognitive functioning). Procedures for this group are described below. Only non-pregnant women will participate in the greater-intensity activities for reasons related to participant burden. We will select the non-pregnant subset of women for these outcomes through the household listing based on proportion-to-size principles (and by considering costs to minimize travel by limiting the smallest sample size per village to at least 10 participants). Pregnant women will only be excluded from the three tests included as greater-intensity activities and anthropometric measurements, they are still eligible for hemoglobin measurements and the interview.

Everyone involved in the study (data collectors, the principal investigator, program implementers, project managers, etc.) except two staff members, will be blinded to who is in the treatment and control clusters.

### Data Collection & Measurement

All participants (*n* = 2000 in treatment and *n* = 2000 in control clusters) will first undergo a point-of-care hemoglobin test to assess anemia status, followed by biometric assessments (height and weight), and a one-on-one survey interview to assess demographic information, psychosocial factors, and anemia-related behaviors. In order to minimize interview time, a planned missingness design was used to create four shortened versions of the survey. All versions of the survey contain the main study outcomes, certain secondary outcomes, and basic demographics. Participants will be randomly assigned to receive one of the six versions. This process will significantly reduce participant burden (in comparison to having all participants answer all questions).

The following procedures will be occur for all participants inside their home.

#### Hemoglobin measurements

We will obtain hemoglobin levels from all participants, through point-of-care hemoglobin tests, using a HemoCue photometer (in line with India’s National Family Health Survey methodology). This instrument provides hemoglobin levels immediately and accurately [24].

#### Interview

All participants will respond to a structured interview administered by a member of the local data collection team. This survey will measure self-reported IFA consumption and anemia status, as well as other secondary outcomes: knowledge, attitudes, and perceptions among participants; social norms; gender norms; mental health (via the CES-D scale); quality of life (via the SF-12); and diet (via the MDD-W questionnaire).

As mentioned, a smaller subset of non-pregnant women (*n* = 150 in each arm) will be randomly selected to provide further data on physical activity, work capacity, and socio-cognitive functioning. These measurements will be collected in a nearby community facility.

#### Work capacity

The Queens College Step Test assesses aerobic fitness [25]. The participant steps up and down on the 16.25-in. (41.3 cm) high platform at a rate of 22 steps per minute (88 beats per minute), assisted by the use of a metronome to maintain the right speed. Participants use a four-step cadence, ‘up-up-down-down’ for 3 min, and heartbeat is assessed at five points: *t*_*0*_ to *t*_*4*_*,* corresponding to the beginning (before starting the step test), at the 1-, 2-, and 3-min marks, and then at the end (1 min after completing the step test). During our pilot study, we learned that the 16.25 in. height was too high for our sari-wearing participants and that a 12-in. height was found to be ideal. We will use this (12-in.) height in our study. Although using this lower height will not make our study readings directly comparable with other published studies, it will help us address our study objective (to compare longitudinally and across treatment-control arms).

#### Socio-cognitive functioning

We will also measure cognitive functioning within this sub-cohort through attention and working memory tasks. We will use the Simon Task and a Simple Reaction Time test to measure attention. We will also use a Corsi Blocks task and a Word Span test to assess working memory. To account for low computer literacy, these tests include both computer and non-computerized tests. Manual and computer based cognitive testing has successfully been carried out in several low and middle income countries in both rural and peri-urban settings including India [26–28]. All four tests will be administered by data collection staff who have been trained by the PI using a framework designed by an expert in cognitive testing.

#### Physical activity

Participants in the sub-cohort will be asked to wear an ActivPAL (PAL Technologies, LTD; Glasgow, UK) for three consecutive days to establish baseline measures of daily reclining, sitting, standing, and walking. The ActivPAL is small (53 × 35 × 7 mm), light-weight (15 g) and is attached to the mid-thigh.

### Statistical power and sample size calculations

We assume a 7% reduction in anemia (from 47 to 40%), which is a lower effect size than typically found, alpha level of .05 with 80% power, the required sample size is 787 per arm [29, 30]. Further assuming a design effect of 2.0 to account for clustering effects within villages, the required sample size with 20% loss to follow up is *N* = 1968 per arm, which will be rounded up to 2000 per arm for a total of *N* = 4000 across the treatment and control arms at baseline.

The more intense assessments consisted of three components – cognitive functioning, physical activity, and step test. We calculated the required sample size for the cognitive functioning component, assuming that cognitive functioning would improve by 16% and we assumed that ICC would not be an issue. This required a sample size of 288, which we rounded up to 300. Sample size calculations for the other two components, ActivPal and Step Test, were not done separately because these tests are administered to the same sample as the cognitive test. The sample size matrix can be found in Table 3.

**Table 3:** Sample Size Matrix

Study component	Intervention difference	Power	Alpha	Design effect	Required N	Rounded N
Main trial	7% anemia	80%	0.05	2	3936	4000
Cognitive functioning	16% more functioning	80%	0.05	1	288	300

### Statistical analysis

At baseline, we will conduct a series of bivariate tests across treatment and control arms. While randomization should ensure baseline matching, this is an effective technique to find any baseline difference. If any are found, they will be controlled for in any midline analysis. If we are unable to find baseline differences at midline, we will conduct a series of bivariate tests, including chi-square tests in which the binary outcome of anemia status at midterm is compared across treatment and control arms. This will be repeated at end line. Additionally, we will test the hypothesis that the treatment group will display a greater increase in hemoglobin count in comparison to the control group, supplemented with a difference-in-difference analysis in which change in hemoglobin levels between midline and baseline in the treatment group will be compared with corresponding changes in the control group. Thus, if there are differences across arms at baseline, we can still compare any observed change across arms. We will conduct another similar analysis at end line, using multivariate analysis of variance (MANOVA) techniques to uncover non-linear trends in the data. We will also evaluate the ability of the intervention to reduce anemia among pregnant versus non-pregnant women, as well as younger versus older women.

The primary analysis for hypothesis 1 will follow an intent-to-treat assumption. We will analyze data at the individual level, adjusting for clustering (at the village and cluster level), using generalized estimating equations (GEE). We will conduct a similar analysis through hierarchical linear modeling to account for village-level clustering effects, but this time with hemoglobin levels (continuous variable) as the dependent variable. In our MANOVA, time, treatment, and time x treatment will serve as the independent variables.

To test the hypothesis that social norms are the primary mediator between the intervention and change in hemoglobin levels, we will use structural equation models (SEM). The mediation analysis will include the secondary outcomes in the pathway between exposure to the intervention and hemoglobin level change.

Subsequent analyses will include other variables as outcomes, including those pertaining to physical activity; work capacity; quality of life; cognitive functioning; mental health; and psychosocial factors, including knowledge, attitude, normative beliefs, IFA intentions, and IFA use.

### Ethics and dissemination

#### Research ethics approval

This study was approved by the George Washington University Institutional Review Board (IRB) and Sigma Science and Research, an IRB located in New Delhi, India. The study was also reviewed and approved by Indian Council for Medical Research’s (ICMR’s) Health Ministry’s Screening Committee (HMSC). Any changes to study protocol will be communicated with these regulatory entities for approval immediately.

### Participant consent and confidentiality

Informed consent will be obtained in Oriya by local data collectors from DCOR Consulting. Data collectors will read the consent document to participants, who will then sign to indicate their consent. Participants under the age of 18 are required to obtain the written permission of one parent or legal guardian. All data from participants will be de-identified by the local principle investigator and stored in secure, password-protected computers that only the study team and its affiliates have access to.

### Dissemination

In addition to disseminating our work at conferences and in peer-reviewed academic journals, we will disseminate findings through multi-media channels as peer-reviewed findings get published. We will also update policy makers and stakeholders continuously with progress reports and newsletters. Finally, we will report findings back to the community where the research takes place after each data collection phase.

### Risk mitigation plan

This is a low risk study so we do not anticipate serious adverse events. However, we will take precautions to ensure the safety of participants. Data collectors will be trained on HemoCue testing, how to communicate hemoglobin levels and anemia status to women following each test, and how to properly dispose of all HemoCue testing materials. While there will not be an independent assessment team to evaluate overall impact, we have put together an independent data safety and monitoring board (DSMB) to assess the outcomes on an ongoing process in order to ensure that the intervention does not inadvertently harm any participants.. The independent DSMB will review any serious adverse events and make recommendations for informing the IRB or stopping the study altogether. The DSMB includes researchers from India and the United States who will meet quarterly to review and discuss initial results from the study and any potential risks for participants.

## Discussion

While anemia has been a public health concern in India for decades, to our knowledge, no intervention has used a social-norms based model to encourage IFA use and iron rich food consumption. Our formative research findings showed the importance of shifting social norms among WRA and their primary referent groups (e.g., their husbands and mothers-in-law). We developed the T4 approach based on the formative research findings and the theory of normative social behavior. The longitudinal evaluation of the RANI T4 approach will both evaluate the efficacy of a norms-based intervention to increase uptake of IFA and iron-rich food as well as investigate the role of social norms as a mediator in anemia-related behavior change.

We will encourage input from stakeholders throughout the implementation and evaluation of the RANI project. The promotion of IFA uptake is in line with the agenda of the Government of Odisha [16], the National Iron Plus Initiative of India [8], the WHO Sustainable Development Goals [7], and the Anemia Mukt Bharat [31]. The findings from this study can provide evidence-based methods to reduce anemia within the state of Odisha through an innovative social-norms lens. Findings may be applicable to other rural areas of India and South Asia.

The intervention design and implementation is based on tested theory and extensive formative research with the target community. A cluster-randomized controlled design improves internal validity. We minimized the risk of contamination between the intervention and control conditions by including a geographical buffer of one or two villages in between treatment and control clusters. As village clusters were randomly selected for inclusion into the study, and then randomly assigned into treatment and control arms, both between- and within- cluster variation should be similar across both treatment and control groups. The inclusion of a control group further improves the internal validity of this study as it allows for the consideration of secular trends, which is particularly important in Odisha, India where ongoing efforts to reduce anemia by other groups can introduce history bias.

Additionally, a longitudinal evaluation design provides a better understanding of hemoglobin level changes with IFA use. The use of three time points also allows for the investigation of non-linear trends in hemoglobin levels after exposure to the intervention.

Blinding at two levels may strengthen the quality of our data. At the first level, the intervention implementers have been blinded to which villages are selected for data collection. This will minimize bias in implementation, allowing for our selected sample for data collection to be representative of all the villages that received the RANI treatment. At the second level, the data collectors will be unaware of which villages are selected for treatment and control. This will also minimize any potential recording bias as the data collectors will not know if they are currently collecting data from an individual in the treatment or control arm.

### Limitations

To our knowledge, this is the first study that tests the effects of a norms-based intervention to improve IFA demand among WRA in India through a randomized controlled trial. As a result, we use a “kitchen sink” approach, in which we assess the overall impact of all intervention components, without distinguishing which component may have been most effective. However, we will measure exposure to components of the intervention as well as collect monitoring and processes evaluation data to understand the effects of specific intervention activities to the extent possible.

Additionally, this is primarily a demand-side intervention. Our formative research shows that access to IFA is not a major barrier to use, but if our intervention is successful, an increase in demand may impact supply. If supply chain problem occurs, changes in social norms may have limited impact. Therefore, we will be monitoring stock-outs throughout the intervention and we will use monitoring and evaluation data at the block level to advocate for additional supply if/when demand increases. The study length supports an evaluation of the short-term effectiveness of the intervention but does not evaluate longer-term sustainability of the social norms or behavior changes.

The current Indian guidelines suggest IFA supplementation once a week for non-pregnant women and once a day for pregnant women in their second and third trimester (both comprised of 60 mg elemental Iron + 500 mcg Folic Acid) [16]. Abdominal pain is commonly reported among women who take IFA daily, but less frequently in women who take IFA weekly [32]. As pregnant women experience the side effects associated with daily dosage, the reputation of IFA may dampen and reduce demand. Previous studies show that women who experience side effects, such as abdominal pain, or believe that they are caused by IFA are less likely to adhere to IFA than those who do not [33, 34]. Dosage regimen also influences absorption of iron; clinical studies have shown that IFA with ≥60 mg iron administered daily increases hepcidin, subsequently reducing absorption on the next day [35]. However, IFA administered on alternating days yielded approximately twice the amount of iron absorption than daily administration [36].

It is important to note that we only include women in this study’s impact evaluation – understanding normative change among men/husbands would add valuable information to this study. Additionally, the intervention does not target the behaviors of frontline workers; therefore frontline workers may continue practices of distributing IFA only to pregnant women or failing to follow up on IFA adherence.

Additionally, we anticipate that attrition may occur through the course of this study. To minimize any effects to internal validity, the power size calculations were conducted with 20% anticipated attrition. If attrition does occur, we will investigate if systematic differences are observed in baseline among those who drop out of the study.

## Data Availability

Data sharing is not applicable to this article as no datasets were generated or analyzed during the current study. The dataset(s) that will come out of this study will be available in the Gates Open Access repository.

## Abbreviations

IFA: Iron-folic acid
RCT: Randomized controlled trial
WHO: World health organization
WRA: Women of reproductive health
NIPI: National iron plus initiative
RANI: Reduction in anemia through normative innovations
SHG: Self-help groups
TNSB: Theory of normative social behavior
